# Bioaugmentation with Plant Growth-Promoting Rhizobacteria Alleviates Chromium and Salt Stress in Rice Through the Improvement of Physiology, Ion Homeostasis, and Antioxidant Defense

**DOI:** 10.3390/microorganisms13071462

**Published:** 2025-06-24

**Authors:** Muhammad Abdus Sobahan, Nasima Akter, Muhammad Manjurul Karim, Md. Muzahidul Islam Badhon, Shakila Nargis Khan, Samiul Alam, P.V. Vara Prasad, Mirza Hasanuzzaman

**Affiliations:** 1 Department of Agronomy, Faculty of Agriculture, Sher-e-Bangla Agricultural University, Sher-e-Bangla Nagar, Dhaka 1207, Bangladesh; sobahansard@bou.ac.bd (M.A.S.); samiulalam-1707649@sau.edu.bd (S.A.); 2 School of Agriculture and Rural Development, Bangladesh Open University, Gazipur 1705, Bangladesh; 3 Agronomy Division, Bangladesh Rice Research Institute, Gazipur 1701, Bangladesh; nasima.agro@brri.gov.bd; 4 Department of Microbiology, University of Dhaka, Dhaka 1000, Bangladesh; manjur@du.ac.bd (M.M.K.); muzahidul.badhon@icddrb.org (M.M.I.B.); shakila@du.ac.bd (S.N.K.); 5 Department of Agronomy, Kansas State University, Manhattan, KS 66506, USA; vara@ksu.edu

**Keywords:** antioxidant defense, ion homeostasis, osmotic stress, oxidative stress, PGPR, toxic metals

## Abstract

Salinity and heavy metal stress significantly reduce agricultural productivity in arable lands, particularly affecting crops like rice (*Oryza sativa* L.). This study aimed to evaluate the efficacy of heavy metal-tolerant plant growth-promoting rhizobacteria (HMT-PGPR) in mitigating the harmful effects of salt (NaCl), chromium (Cr), and combined NaCl + Cr stress on rice plants. Two pre-isolated and well-characterized heavy metal-tolerant epiphytic (*Ochrobactrum pseudogrignonense* strain P14) and endophytic (*Arthrobacter woluwensis* strain M1R2) PGPR were tested. The LSD test (*p* ≤ 0.05) was used to assess the statistical significance between treatment means. Stresses caused by NaCl, Cr, and their combination were found to impair plant growth and biomass accumulation through mechanisms, including osmotic stress, oxidative damage, ionic imbalance, reduced photosynthetic pigment, lowered relative water content, and compromised antioxidant defense systems. Conversely, inoculation with HMT-PGPR alleviated these adverse effects by reducing oxidative stress indicators, including malondialdehyde (MDA), hydrogen peroxide (H_2_O_2_) content and electrolyte leakage (EL) and enhancing plant growth, osmolyte synthesis, and enzymatic antioxidant activity under single- and dual-stress conditions. The application of HMT-PGPR notably restricted Na^+^ and Cr^6+^ uptake, with an endophytic *A. woluwensis* M1R2 demonstrating superior performance in reducing Cr^6+^ translocation (38%) and bioaccumulation (42%) in rice under dual stress. The findings suggest that *A. woluwensis* effectively mitigates combined salinity and chromium stress by maintaining ion homeostasis and improving the plant’s antioxidant defenses.

## 1. Introduction

Rapid changes in the environment and rising sea levels due to the melting of ice caps and glaciers are exacerbating seawater intrusion into coastal agricultural lands [1]. Salinity poses a significant challenge to cultivable lands worldwide, with approximately 7% of global and 20% of irrigated lands currently affected by excessive salt [2,3]. Projections indicate that over 50% of cultivable land could face salinization by 2050 [4]. Soil salinity results from the accumulation of soluble salts [5] that adversely affect plants by disrupting ionic balance, inducing nutrient deficiencies, damaging lipid membranes, reducing photosynthetic rates, and generating osmotic and oxidative stress through reactive oxygen species (ROS) [6,7,8].

Heavy metals (HMs) are another major environmental concern, negatively impacting crop growth and productivity due to their toxicity and bioaccumulation potential [9,10]. Chromium, Cr(VI), one of the most toxic HMs, poses significant threats to biological systems [11] due to its higher electronegativity [12,13]. Being highly soluble in soil and water, Cr(VI) is mobile and permeable through biological membranes [14], disrupting enzyme activities and generating ROS that can damage proteins, lipids, and DNA [15].

Chromium contamination in soils is a critical yet understudied issue, particularly in rapidly industrializing regions like Bangladesh, where industrial effluents and urbanization have led to widespread heavy metal pollution. Unlike salinity, which primarily reduces crop productivity, chromium (especially Cr(VI)) poses severe health risks, including carcinogenicity, and accumulates in the food chain. Its presence in agricultural soils near industrial hubs exacerbates stress conditions, disrupting nutrient uptake and increasing oxidative damage in plants. In Savar, Dhaka, soil Cr levels have been reported between 100 and 1200 mg L^−1^, far exceeding the maximum permissible limit of 100 mg L^−1^ [16]. Due to its persistence and toxicity, Cr contamination demands urgent attention, with sustainable solutions like rhizoremediation offering potential mitigation. Thus, while salinization is a major agricultural challenge, Cr pollution represents a unique and pressing threat to both ecosystem health and human safety, warranting further research, especially in combined stress scenarios.

Plants combat Cr toxicity by producing osmolytes and antioxidants to maintain ROS homeostasis [17,18,19]. Rice (*Oryza sativa* L.), a staple cereal crop, is particularly sensitive to both salinity and HM stress. Elevated Na*^+^* and Cr levels significantly hinder rice growth, development, and yields [20,21]. Enhancing rice tolerance to these stressors is critical for food security. One promising approach involves the use of plant growth-promoting rhizobacteria (PGPR), beneficial soil microbes that promote plant growth and development through various mechanisms [22,23]. The PGPR aids stress tolerance by solubilizing nutrients (e.g., phosphorus and potassium), fixing nitrogen, producing phytohormones, 1-amino cyclopropane-1-carboxylate (ACC) deaminase, siderophores, exopolysaccharides (EPS), and by competing against pathogens [24,25].

Several PGPR species, including *Arthrobacter*, *Agrobacterium*, *Azotobacter*, *Azospirillum*, *Alcaligenes*, *Bacillus*, *Bradyrhizobium*, *Burkholderia*, *Enterobacter*, *Ochrobactrum*, *Rhizobium*, *Pseudomonas*, *Klebsiella*, and *Chromobacterium*, exhibit resilience to salinity, metals, and other abiotic stresses, enhancing plant growth under adverse conditions [26,27]. For instance, *Ochrobactrum pseudogrignonense* increased drought tolerance in black gram (*Vigna mungo* L.) and garden pea (*Pisum sativum* L.) plants by producing ACC deaminase, which regulates ethylene levels in these plants [28], while *Arthrobacter woluwensis* AK1 significantly improved soybean (*Glycine max* L. Merril) growth with or without salinity stress by producing siderophores, gibberellic acid (GA), and indole-3-acetic acid (IAA) [29]. In this regard, it is crucial to consider the possibilities of heavy metal-tolerant PGPR (HMT-PGPR) to enhance growth and biochemical parameters in rice plants affected by salinity and Cr toxicity. Recent studies suggest that PGPR inoculation improves plant growth and stress tolerance in saline and metal-contaminated soils [30]. However, the combined effects of HMT-PGPR on rice growth under NaCl- and Cr-contaminated conditions remain unexplored. Therefore, the study aimed to: (i) evaluate the effects of HMT-PGPR on the growth, physiological, and biochemical attributes of rice plant under salinity (NaCl), Cr, and combined NaCl + Cr stress, and (ii) investigate the role of HMT-PGPR in regulating Cr translocation and bioaccumulation under these stress conditions.

## 2. Materials and Methods

### 2.1. Plant Materials and Growth Conditions

A pot trial was performed at Sher-e-Bangla Agriculture University, Dhaka, Bangladesh. The soil used in this study was collected from a farmer’s field. The soil pH and electrical conductivity (EC) were analyzed using a pH meter (HACH HQ40d, Hach, Loveland, CO, USA) in a 1:10 ratio and an EC meter (HANNA, Hanna instruments, Smithfield, RI, USA), respectively. Soil organic carbon (OC), total N, available P, available S, and exchangeable K were determined by the potassium dichromate wet oxidation, micro Kjeldahl digestion, molybdenum blue colorimetry, turbidimetric, and ammonium acetate method, respectively. The soil had pH (5.4), EC (0.14 dS m^−1^), OC (0.92%), total N (0.09%), available P (8.7 µg g^−1^), available S (21.5 µg g^−1^), available Cr (1.5 mg kg^−1^), and exchangeable K (0.17 meq 100 g^−1^ soil). The zinc-enriched rice variety BRRI dhan100 was used in this study. Non-drained plastic pots with a diameter of 25 cm, height of 24 cm, and volume of 14 L were filled with 12 kg of air-dried soil. Pots were fertilized with Bangladesh Rice Research Institute [31] recommended fertilizers dose (urea, triple super phosphate, muriate of potash, gypsum, and zinc sulphate at the rates of 138, 51, 63, 60, and 4 kg ha^−1^, respectively).

### 2.2. Preparation of Bacterial Inocula, Treatments, and Design

The bacterial strains (*O. pseudogrignonense*, strain P14 and *A. woluwensis*, strain M1R2) with GenBank accession numbers PQ809998 and PQ809999, respectively, were obtained from the Department of Microbiology, University of Dhaka, Bangladesh. Briefly, a single pure colony of each strain, pre-cultured on Tryptic Soy Agar (TSA) plates, was inoculated into 100 mL of Tryptic Soy Broth (TSB) and incubated in a shaking incubator (37 °C, 120 rpm) until the mid-exponential growth phase (OD_600_ at 0.5) [U-2910 Spectrophotometer, Hitachi, Tokyo, Japan] was reached. Cells were harvested by centrifugation at 8800× *g* (Tomy MX-305 refrigerated centrifuge, Japan) for 5 min at 4 °C, and the resulting pellet was washed twice and re-suspended in 400 mL of phosphate-buffered saline (PBS; pH 7.4) to achieve a uniform cell suspension. The roots of 36-day-old rice seedlings were dipped separately in the bacterial suspensions (10^8^ CFU mL^−1^) and kept at normal temperature for 1 h. For the second inoculation, 10 mL of PBS solution-washed bacterial cells were added to the soil in the pot at 21 days after transplanting (DAT). Bacterial treatments (uninoculated, P14, and M1R2) were used under different conditions (control, NaCl, K_2_Cr_2_O_7_, and NaCl + K_2_Cr_2_O_7_). Each treatment comprised three replications by using a completely randomized design (CRD). For stress treatments, sodium chloride (NaCl, 80 mM) and potassium dichromate (K_2_Cr_2_O_7,_ 500 µM) were applied at 21 DAT. The NaCl and K_2_Cr_2_O_7_ concentrations were gradually increased by 25 mM and 100 µM, respectively, until the required salinity (80 mM) and chromium stress (500 µM) were reached. Plants were irrigated at 3-day intervals with NaCl and Cr solution for 7 weeks. In detail, salt-stressed plants received 25, 40, 50, 65, and 80 mM NaCl solution for 2, 2, 1, 1, and 1 week, respectively. For Cr-stressed plants, they received 100, 200, and 500 of K_2_Cr_2_O_7_ solution for 3, 3, and 1 week, respectively. Data on various morphophysiological and biochemical parameters were collected at 65 DAT.

### 2.3. Measurements of Crop Growth Attributes

The plants were harvested from the pot soil and washed by tap water. The plants were separated into roots and shoots. Then, shoot and root length were measured by measuring the scale and weighed to record fresh weight (FW). Dry weights (DW) of each part were recorded after oven-drying at 70 °C for 72 h. Percentage increase of dry weight was calculated using the following formula: Dry weight of stressed plants with inoculation − Dry weight of stressed plants/Dry weight of stressed plants × 100. The leaf area was measured after treatment exposure. A LI-COR 3100 digital leaf area meter (LI-COR, Inc., Lincoln, NE, USA) was used to estimate the leaf area.

### 2.4. Calculation of NaCl and Cr Stress Tolerance Index

The NaCl and Cr stress tolerance index (STI) was calculated using the following formulae [32]:

STI = Dry weight of stressed plants/Dry weight of control plant × 100

STI = Dry weight of stressed plant with inoculation/Dry weight of control plant × 100

### 2.5. Measurements of Physiological and Biochemical Attributes

#### 2.5.1. Determination of Relative Water Content

The leaf relative water content (RWC) was measured by the method of Sairam et al. [33]. The expanded leaves were cut and instantly weighed to measure FW. The leaf samples were then soaked in Petri dishes containing deionized water and incubated for 4 h. Then, the leaves were dried on paper and weighed to collect the turgid weight (TW). Thereafter, the leaves were dried at 70 °C for 48 h to estimate DW. The leaf RWC was computed following the equation: RWC (%) = (FW − DW)/(TW − DW) × 100.

#### 2.5.2. Determination of Proline

Proline was determined following the method described by Bates et al. [34]. Fresh leaf tissue (0.25 g) was homogenized in 3% (*w*/*v*) aqueous sulfosalicylic acid using a mortar and pestle. The homogenate was then centrifuged at 12,000× *g*. An aliquot of 2 mL of the resulting supernatant were mixed with 2 mL of glacial acetic acid and 2 mL of acid ninhydrin reagent. The mixture was incubated in a water bath at 95 °C for 1 h. After incubation, the reaction tubes were cooled on ice. The chromophore was extracted with toluene by thorough mixing, followed by vortexing. The absorbance of the toluene phase was measured at 520 nm using a spectrophotometer. A standard curve was prepared using *L*-proline to quantify the proline concentration.

#### 2.5.3. Measurement of Chlorophyll Fluorescence

Chlorophyll (Chl) fluorescence was measured by a PEA (plant efficiency analyzer) portable Chl fluorometer (Hansatech Instruments Ltd., Norfolk, UK). The minimal fluorescence (F_o_) was monitored in an imitated dark condition. Fifteen minutes later, the maximal fluorescence (F*_m_*) and variable fluorescence (F*_v_*) were measured by a slight pulse (3000 mmol m^−2^ s^−1^). F*_v_*/F*_m_* was computed following the formula: F*_v_*/F*_m_* = (F*_m_* − F_o_)/F*_m_* where F*_v_* = variable fluorescence.

#### 2.5.4. Determination of Photosynthetic Pigments

Leaf (100 mg, FW) was completely extracted with 10 mL of acetone (80%). The absorbance of the extract was monitored at 645, 663, and 470 nm, respectively, for Chl *a*, Chl *b*, and carotenoid (Car) content. The following equations were used for calculation [35]: Chl *a* = 11.75 A_663_ − 2.350 A_645_, Chl *b* = 18.61 A_645_ − 3.960 A_663_, Chl (*a* + *b*) = Chl *a* + Chl *b*, and total Car = (1000A_470_ − 2.270 Chl *a* − 81.4 Chl *b*)/227.

#### 2.5.5. Determination of Total Na^+^ and K^+^

The sodium (Na^+^) and potassium (K^+^) were measured following the method described by Yoshida et al. [36]. Roots and shoots of the treated rice plants were dried at 70 °C and ground using a mechanical grinder. The powdered samples were then sieved through a 2 mm mesh. A 100 mg portion of each sample was placed into a conical flask, and 10 mL of 1N HCl were added, ensuring the sample was fully submerged without shaking. The samples were left to stand overnight at room temperature. The extracts were then filtered and diluted to a final volume of 100 mL. The concentrations of Na^+^ and K^+^ were determined using a flame photometer (Flame Photometer 410, Sherwood Scientific, Cambridge, UK).

#### 2.5.6. Cr Analysis in Shoot, Root, and Soil

Dried and ground plant samples (0.5 g) were mixed with 5 mL of 69% nitric acid (HNO_3_) and left overnight for pre-digestion. Subsequently, 2 mL of 60% perchloric acid (HClO_4_) were added, and the mixture was digested on a hot plate at 120 °C for 4.5 h. The digested sample was then diluted to 25 mL with deionized water in a volumetric flask. The concentration of Cr in the solution was determined using an atomic absorption spectrophotometer (AA-7000, Shimadzu, Kyoto, Japan), following the method of Campbell and Plank [37].

The translocation factor (TF) and bioaccumulation factor (BAF) for Cr were calculated using the following formulas, as described by Shahabivand et al. [38]:

TF = Cr content in the shoot (mg kg^−1^)/Cr content in the roots (mg kg^−1^)

BAF = Cr content in the shoot (mg kg^−1^)/Cr content in soil (mg kg^−1^)

### 2.6. Determination of Oxidative Stress Indicators

#### 2.6.1. Determination of Lipid Peroxidation

Malondialdehyde (MDA) content (an indicator of lipid peroxidation) in the leaf tissue was measured using the thiobarbituric acid (TBA) method described by Madhava Rao and Sresty [39]. Fresh leaf tissue (0.5 g) was homogenized in 2 mL of 5% trichloroacetic acid (TCA) and centrifuged at 12,000× *g* for 15 min. One milliliter of the supernatant was then mixed with 2 mL of 20% TCA containing 0.5% TBA. The mixture was incubated in a hot water bath for 25 min and subsequently cooled to room temperature. The absorbance of the solution was recorded at 532 nm and 600 nm using a spectrophotometer. The MDA concentration was calculated using an extinction coefficient of 155 mM^−1^ cm^−1^.

#### 2.6.2. Determination of Hydrogen Peroxide

The hydrogen peroxide (H_2_O_2_) content in leaf tissues was determined following the method described by Yu et al. [40]. Fresh leaf tissue (0.5 g) was homogenized in 5% TCA, and the extract was centrifuged at 12,000× *g* for 12 min. The resulting supernatant was mixed with 10 mM of potassium phosphate buffer (pH 7.0) and 1 mM of potassium iodide (KI). The absorbance of the reaction mixture was measured at 390 nm using a spectrophotometer. The concentration of H_2_O_2_ was calculated from a standard curve prepared with known concentrations of H_2_O_2_.

#### 2.6.3. Determination of Electrolyte Leakage

Electrolyte leakage (EL) was measured following the method described by Dionisio-Sese and Tobita [41]. Fresh leaf tissue (0.2 g) was cut into small pieces and placed in test tubes containing 10 mL of distilled water. The samples were incubated in a water bath at 40 °C for 1 h. After cooling to room temperature, the initial electrical conductivity (EC_1_) of the solution was measured using a conductivity meter (HI-993310, Hanna Instruments, Smithfield, RI, USA). The samples were then autoclaved at 121 °C for 40 min and allowed to cool to room temperature. The final electrical conductivity (EC_2_) was then measured. Electrolyte leakage was calculated using the following formula: EL (%) = (EC_1_/EC_2_) × 100.

### 2.7. Enzyme Extraction and Estimation of Free Protein

Enzymes were extracted following the method of Hasanuzzaman et al. [42]. Fresh leaf tissue (0.5 g) was homogenized in an ice-cold mortar and pestle using an extraction buffer containing 1 mM of *L*-ascorbic acid (AsA), 100 mM of KCl, 50 mM of potassium phosphate buffer (pH 7.0), 5.0 mM of *β*-mercaptoethanol, and 10% (*w*/*v*) glycerol. The homogenate was centrifuged at 11,500× *g* for 15 min at 4 °C. The total protein content in the enzyme extract was determined using the Bradford method [43], with bovine serum albumin (BSA) used to prepare the standard curve. The absorbance of the extracted aliquots was measured at 595 nm using a spectrophotometer.

### 2.8. Estimation of Antioxidant Enzyme Activity

Peroxidase (POD; EC 1.11.1.7) activity was determined following the method of Hemeda and Klein [44]. The reaction mixture contained 0.5 M of potassium phosphate (K-P) buffer (pH 7.0), 0.05% guaiacol, 30 mM of H_2_O_2_, and the enzyme extract. The activity was measured at 470 nm due to guaiacol oxidation, using an extinction coefficient of 26.6 mM^−1^ cm^−1^.

Catalase (CAT; EC 1.11.1.6) and glutathione-*S*-transferase (GST; EC 2.5.1.18) activities were assayed according to Hasanuzzaman et al. [42]. This CAT activity was determined at 240 nm by mixing the enzyme extract with 50 mM of K-P buffer (pH 7.0) and 15 mM of H_2_O_2_, using an extinction coefficient of 39.4 mM^−1^ cm^−1^. The GST activity was measured at 340 nm in a reaction mixture containing 0.25 m of K-P buffer (pH 7.0), 1.5 mM of reduced glutathione (GSH), and 1 mM of 1-chloro-2,4-dinitrobenzene (CDNB), with an extinction coefficient of 9.6 mM^−1^ cm^−1^.

Ascorbate peroxidase (APX; EC 1.11.1.11) activity was estimated following the method of Nakano and Asada [45]. The reaction mixture included 15 mM of K-P buffer (pH 7.0), 0.5 mM of ascorbic acid (AsA), 0.1 mM of EDTA, and H_2_O_2_. The activity was monitored at 290 nm, and the calculation was based on an extinction coefficient of 2.8 mM^−1^ cm^−1^.

Glutathione peroxidase (GPX; EC 1.11.1.9) activity was assayed according to Elia et al. [46], with slight modifications. The reaction medium contained 100 mM of K-P buffer (pH 7.0), 1 mM of EDTA, 1 mM of NaN_3_, 0.12 mM of NADPH, 2 mM of GSH, 1 unit glutathione reductase (GR), 10.5 mM of H_2_O_2_, and the enzyme extract. The activity was calculated using an extinction coefficient of 6.62 mM^−1^ cm^−1^.

Dehydroascorbate reductase (DHAR; EC 1.8.5.1) activity was measured following Nakano and Asada [45]. The assay mixture consisted of 50 mM of K-P buffer (pH 7.0), 2.5 mM of GSH, 0.1 mM of EDTA, and dehydroascorbic acid (DHA). The activity was monitored at 265 nm using an extinction coefficient of 14 mM^−1^ cm^−1^.

### 2.9. Determination of Glyoxalase Enzyme Activity

Glyoxalase I (Gly I; EC 4.4.1.5) activity was determined following the method of Yadav et al. [47]. The reaction mixture contained 100 mM of potassium phosphate (K-P) buffer (pH 7.0), 15 mM of MgSO_4_, 1.7 mM of GSH, 3.5 mM of methylglyoxal (MG), and the enzyme extract. Absorbance was recorded at 240 nm, and activity was calculated using an extinction coefficient of 3.37 mM^−1^ cm^−1^.

Glyoxalase II (Gly II; EC 3.1.2.6) activity was measured according to the method of Principato et al. [48]. The assay solution consisted of 100 mM of Tris-HCl buffer (pH 7.2), 0.2 mM of 5,5′-dithiobis (2-nitrobenzoic acid) (DTNB), 1 mM of *S*-D-lactoylglutathione (SLG), and the enzyme extract. Absorbance was measured at 412 nm, and activity was calculated using an extinction coefficient of 13.6 mM^−1^ cm^−1^.

### 2.10. Statistical Analysis

Data were obtained from three replicates and expressed as mean ± standard deviation (SD). One-way analysis of variance (ANOVA) was performed using CropStat 10 software. The least significant difference (LSD) test at *p* ≤ 0.05 was used to determine statistical significance among treatment means.

## 3. Results

### 3.1. Effect of HMT-PGPR Inoculation on Plant Growth and Biomass Accumulation

**Figure 1:**
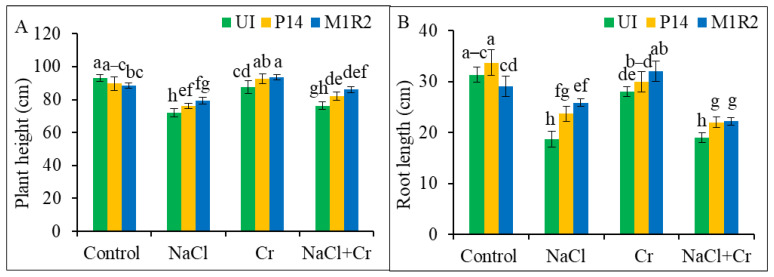
Effect of bacterial inoculation on plant height (**A**) and root length (**B**) of rice plants (65 DAT) under NaCl, Cr, and combined NaCl + Cr stress conditions. Each column represents the mean ± standard deviation (SD) of three biological replicates. Error bars indicate the least significant difference (LSD) at a 5% level of significance. Different letters indicate statistically significant differences between treatments at *p* < 0.05, UI = Uninoculated.

**Figure 2:**
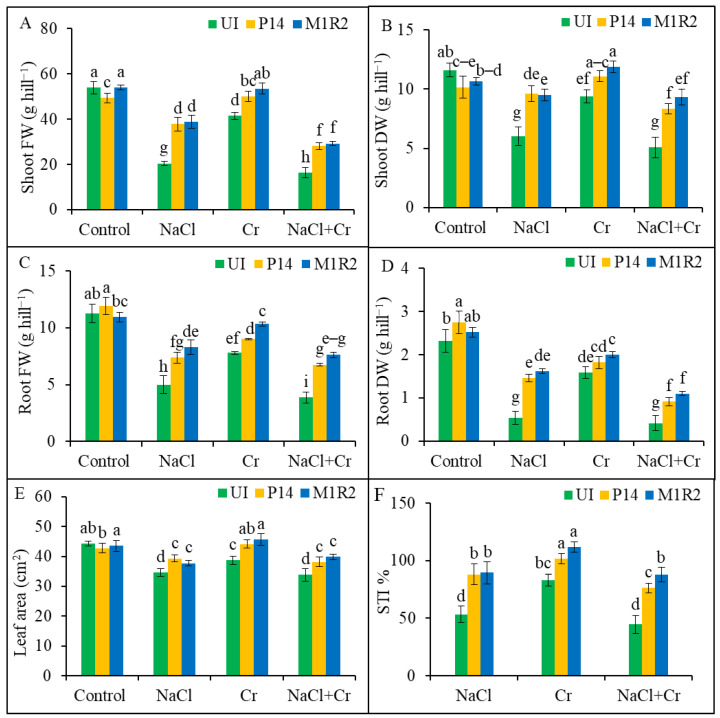
Effect of bacterial inoculation on shoot and root biomass, leaf area, and stress tolerance index (STI) of rice plants (65 DAT) under NaCl, Cr, and combined NaCl + Cr stress conditions. Parameters shown include: (**A**) Shoot fresh weight (FW), (**B**) Shoot dry weight (DW), (**C**) Root fresh weight (FW), (**D**) Root dry weight (DW), (**E**) Leaf area, and (**F**) Stress Tolerance Index (STI). Each column represents the mean ± standard deviation (SD) of three biological replicates. Error bars indicate the least significant difference (LSD) at a 5% level of significance. Different letters indicate statistically significant differences between treatments at *p* < 0.05, UI = Uninoculated.

**Figure 3:**
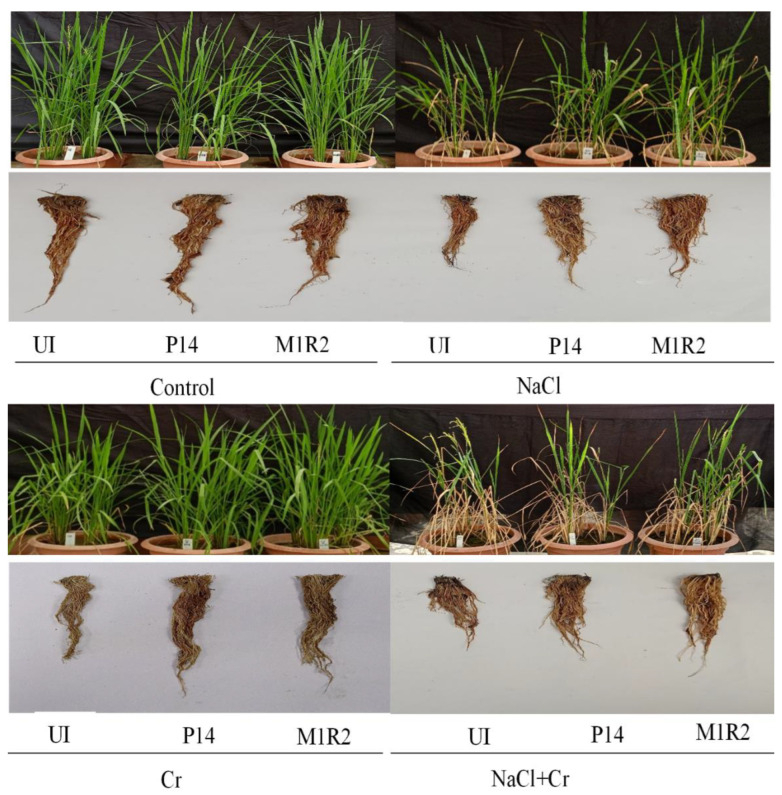
Effect of bacterial inoculation on the growth attributes of rice variety, BRRI dhan100 (65 DAT) under NaCl (80 mM), Cr (500 µM), and combined NaCl + Cr stress conditions. Visual differences in plant growth reflect the impact of stress and the mitigating effects of PGPR inoculation, UI = Uninoculated.

Stresses imparted by NaCl, or Cr, and a combined stress of NaCl + Cr significantly inhibited the growth parameters of rice plants (Figure 1). However, inoculation with the bacterial strains P14 (epiphytic) and M1R2 (endophytic) alleviated these detrimental effects, as reflected in Figure 1, Figure 2 and Figure 3. Compared to the control plants, stress conditions notably reduced plant height and root length, with the most pronounced reductions observed under NaCl and NaCl + Cr treatments (Figure 1A,B). Inoculation with P14 or M1R2 significantly improved both plant height and root length under all stress conditions, although no statistical difference was observed between the two strains within the same stress treatment (Figure 1A,B). Thus, epiphytic and endophytic HMT-PGPR effectively promote the growth of rice plants under NaCl, Cr, and NaCl + Cr stress conditions.

Under NaCl, Cr, and NaCl + Cr stress, shoot FW declined by 62, 23, and 48%, respectively, while shoot DW dropped by 48, 56, and 19%, compared to unstressed controls (Figure 2A,B). Inoculation with P14 and M1R2 significantly improved both shoot FW and DW under stress conditions. The enhancements observed were statistically similar between the two strains across all treatments (Figure 2A,B). Likewise, root FW decreased by 56, 31, and 66%, and root DW by 77, 32, and 82%, under NaCl, Cr, and NaCl + Cr stress, respectively, compared to the control (Figure 2C,D). The PGPR treatment mitigated these reductions, with strain M1R2 showing superior performance. In particular, M1R2 increased root FW by 66, 32, and 97%, and root DW by 200, 27, and 165%, under NaCl, Cr, and NaCl + Cr stress, respectively, compared to corresponding uninoculated stressed plants (Figure 2C,D). The effect of M1R2 was calculated by comparing it to uninoculated NaCl-treated plants. Similar methods are used to compare other treatments. These results indicated that PGPR inoculation increase plant biomass that was greater than the non-PGPR treatment under NaCl, Cr, and NaCl + Cr stresses.

Similarly, leaf area was significantly reduced in uninoculated plants under NaCl, Cr, and NaCl + Cr stress by 21, 13, and 24%, respectively (Figure 2E). However, inoculation with P14 and M1R2 improved leaf area under all stress conditions. Specifically, leaf area increased by 14 and 9% (NaCl), 14 and 18% (Cr), and 13 and 18% (NaCl + Cr) with P14 and M1R2, respectively, compared to the uninoculated stressed counterparts (Figure 2E). The differences between the strains were not statistically significant. As shown in Figure 2F, the STI was lowest in uninoculated rice plants under all stress treatments. Plant growth-promoting rhizobacteria inoculation significantly enhanced STI values by 65% (P14) and 68% (M1R2) under NaCl, 22 and 34% under Cr, and 70 and 96% under NaCl + Cr stress, respectively, compared to the corresponding uninoculated stressed plants. Notably, the endophytic strain M1R2 outperformed the epiphytic strain P14 in boosting STI under all stress conditions. The results indicated that the lower the STI value, the greater the toxic effects of NaCl, Cr, and NaCl + Cr on rice plants. These results indicated that the higher the STI values, the lower the toxic effects of NaCl, Cr, and NaCl + Cr on rice plants.

### 3.2. Phenotypic Appearances

The phenotypic appearance of rice plants under various treatments is shown in Figure 3. It is evident that plant growth was visibly hampered under NaCl, Cr, and combined NaCl + Cr stress in the absence of bacterial inoculation. In contrast, inoculation with P14 and M1R2 effectively alleviated the osmotic stress induced by these treatments. As a result, plants treated with the bacterial strains exhibited improved growth and vigor compared to their uninoculated, stressed counterparts (Figure 3).

### 3.3. Effect of HMT-PGPR Inoculation on Photosynthetic Pigments and Chlorophyll Fluorescence

**Figure 4:**
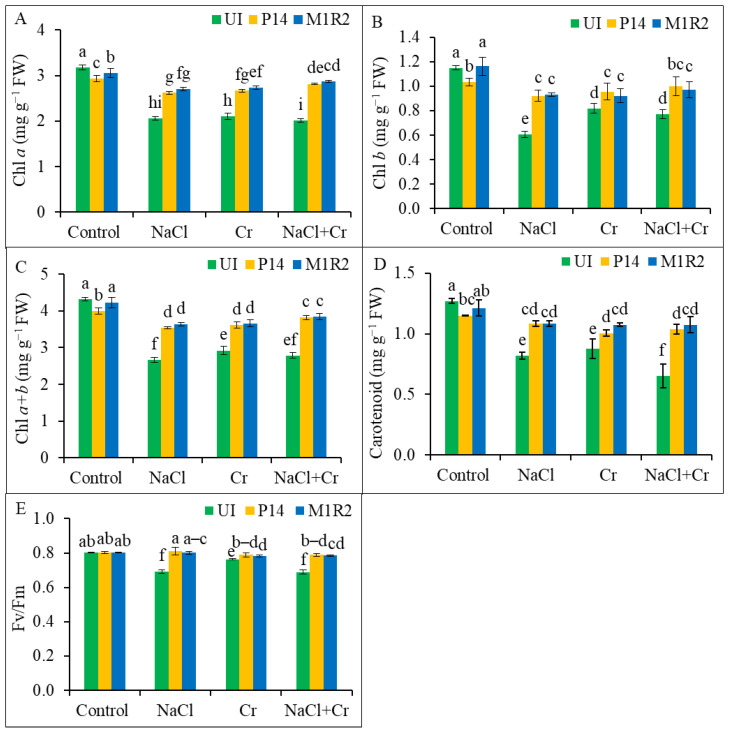
Effect of bacterial inoculation on photosynthetic pigments and chlorophyll fluorescence in rice plants (65 DAT) under NaCl, Cr, and combined NaCl + Cr stress conditions. Panels represent: (**A**) Chlorophyll *a* (Chl *a*), (**B**) Chlorophyll *b* (Chl *b*), (**C**) Total chlorophyll (Chl *a* + *b*), (**D**) Carotenoid content, and (**E**) Maximum quantum efficiency of PSII (F*_v_*/F*_m_*). Each column represents the mean ± standard deviation (SD) of three biological replicates. Error bars indicate the least significant difference (LSD) at a 5% level of significance. Different letters indicate statistically significant differences between treatments at *p* < 0.05, UI = Uninoculated.

Salt (NaCl), Cr, and combined NaCl + Cr stress significantly reduced the levels of Chl *a*, Chl *b*, Chl (*a* + *b*), and Car content in uninoculated rice plants compared to the unstressed control (Figure 4A–D). However, inoculation with PGPR strains P14 and M1R2 markedly improved these photosynthetic pigments under all stress conditions. The strain M1R2 was particularly effective, showing significant increases in pigment content across NaCl, Cr, and NaCl + Cr stress treatments. Specifically, M1R2 enhanced, Chl *a* by 17, 23, and 35%; Chl *b* by 56, 24, and 32%; Chl (*a* + *b*) by 26, 24, and 33%; and Car content by 42, 23, and 76%, respectively, compared to the corresponding uninoculated stressed plants. Notably, under NaCl + Cr stress, M1R2 exhibited the most pronounced effect, significantly restoring Chl *a*, total Chl, and Car contents (Figure 4A–D). These results suggest that HMT-PGPRs enhanced photosynthesis rate through an increase in photosynthetic pigments under NaCl, Cr, or NaCl + Cr stress conditions.

To assess the impact of PGPR on photosynthetic efficiency, the Chl fluorescence parameter F*_v_*/F*_m_*, indicating the maximum quantum efficiency of PS II, was analyzed (Figure 4E). Under NaCl, Cr, and NaCl + Cr stress, uninoculated plants showed reductions in F*_v_*/F*_m_* by 14, 5, and 14%, respectively, relative to the control. In contrast, inoculation with P14 enhanced F*_v_*/F*_m_* by 17, 3, and 15%, and M1R2 increased F*_v_*/F*_m_* by 16, 3, and 14%, respectively, under the same stress conditions. Both strains thus improved PSII photochemical efficiency, with no statistically significant difference between their effects under each stress condition (Figure 4E). These results indicate that inoculation with PGPRs can promote PSII activity (F*_v_*/F*_m_*) in rice plants.

### 3.4. Effect of HMT-PGPR Inoculation on Relative Water Content and Osmoregulation

**Figure 5:**
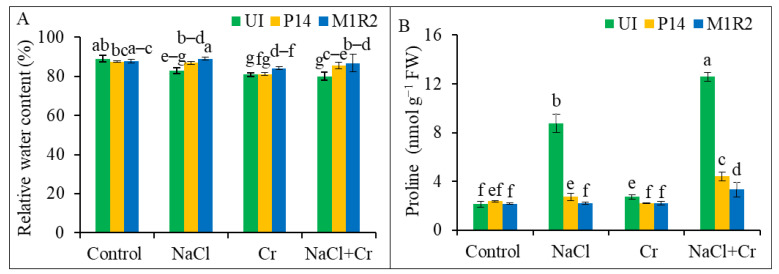
Effect of PGPR inoculation on (**A**) relative water content and (**B**) proline accumulation in rice plants (65 DAT) under NaCl, Cr, and NaCl + Cr stress conditions. Each column represents the mean ± standard deviation (SD) of three biological replicates. Error bars indicate the least significant difference (LSD) at a 5% level of significance. Different letters indicate statistically significant differences between treatments at *p* < 0.05, UI = Uninoculated.

The application of NaCl, Cr, or their combination (NaCl + Cr) significantly reduced the RWC of rice plants by 7, 9, and 10%, respectively, compared to the uninoculated control (Figure 5A). However, inoculation with PGPR strains P14 and M1R2 mitigated these effects and improved RWC under all stress conditions. Notably, M1R2 showed the greatest enhancement, increasing RWC by 8, 5, and 9% under NaCl, Cr, and NaCl + Cr stress, respectively, relative to the corresponding uninoculated stressed plants (Figure 5A). Therefore, the increased water content in PGPR-inoculated plants may be attributed to their longer root systems, which play a key role in enhancing plant defence under stress conditions.

Proline accumulation, a compatible solute playing a key role for osmoregulatory response, significantly increased in rice leaves under all stress treatments. The most pronounced increases were observed under NaCl (309%) and NaCl + Cr (488%) stress compared to the control (Figure 5B). Inoculation with P14 and M1R2 effectively reduced proline accumulation across all stress conditions, indicating improved osmoregulation. Among the strains, M1R2 demonstrated the most substantial reductions in proline content by 75 and 74% under NaCl and NaCl + Cr stress, respectively, when compared to the corresponding stressed controls. Under Cr stress, both strains exhibited statistically similar performance, reducing proline levels by 20% (P14) and 19% (M1R2) (Figure 5B). These results suggest that the lower proline levels induced by PGPR may reflect reduced stress effects of NaCl, Cr, and combined NaCl + Cr treatments in plants.

### 3.5. Effect of HMT-PGPR Inoculation on Oxidative Stress Markers

**Figure 6:**
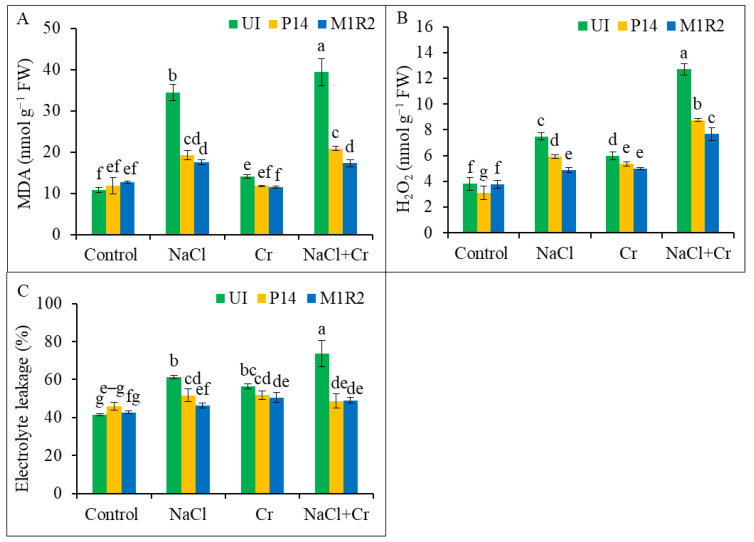
Effect of PGPR inoculation on (**A**) malondialdehyde content, (**B**) hydrogen peroxide content, and (**C**) electrolyte leakage in rice plants (65 DAT) under NaCl, Cr, and NaCl + Cr stress conditions. Each column represents the mean ± standard deviation (SD) of three biological replicates. Error bars indicate the least significant difference (LSD) at a 5% level of significance. Different letters indicate statistically significant differences between treatments at *p* < 0.05. UI = Uninoculated.

The MDA levels in rice leaves, a marker of lipid peroxidation and oxidative stress, were significantly elevated under NaCl, Cr, and NaCl + Cr stress conditions. In uninoculated plants, MDA levels increased by 219, 29, and 264%, respectively, compared to their corresponding controls (Figure 6A). Inoculation with PGPR strains P14 and M1R2 significantly reduced MDA accumulation under all stress conditions. Notably, the endophytic strain M1R2 was more effective than P14, reducing MDA levels by 49 and 56% under NaCl and NaCl + Cr stress, respectively, relative to the corresponding stressed controls. Under Cr stress, both strains performed similarly, showing no statistically significant difference in MDA reduction (Figure 6A). Our results suggest that inoculation with HMT-PGPRs reduces membrane lipid peroxidation, thereby protecting plants from oxidative stress induced by NaCl, Cr, and combined NaCl + Cr treatments.

A significant increase in H_2_O_2_ levels was observed in uninoculated rice plants subjected to NaCl, Cr, or NaCl + Cr stress, indicating oxidative damage and membrane destabilization under these stress conditions (Figure 6B). In contrast, inoculation with PGPR strains P14 and M1R2 significantly reduced H_2_O_2_ accumulation under all stress treatments compared to their respective uninoculated counterparts. Among the strains, endophytic M1R2 consistently outperformed the epiphytic P14 in lowering H_2_O_2_ levels under NaCl, Cr, and NaCl + Cr stress (Figure 6B). Application of PGPR resulted in reduced H_2_O_2_ levels, suggesting their ameliorative role in mitigating ROS accumulation.

Electrolyte leakage in rice leaves increased markedly under all stress conditions, with NaCl, Cr, and NaCl + Cr treatments causing a rise of 48, 36, and 77%, respectively, compared to control plants (Figure 6C). Plant growth-promoting rhizobacteria inoculation significantly reduced EL under all stress conditions. Under NaCl + Cr stress, P14 and M1R2 reduced EL by 34 and 33%, respectively, relative to uninoculated stressed plants. The endophytic M1R2 was particularly effective in reducing EL under NaCl stress, showing the greatest reduction compared to P14. However, under Cr and NaCl + Cr stress, both strains showed statistically similar performance in reducing EL (Figure 6C). Consequently, the lower EL observed in PGPR-inoculated plants suggests that these treatments reduce membrane damage in rice by mitigating ROS accumulation under NaCl, Cr, or NaCl + Cr stress conditions.

### 3.6. Effect of HMT-PGPR Inoculation on Ion Homeostasis

**Figure 7:**
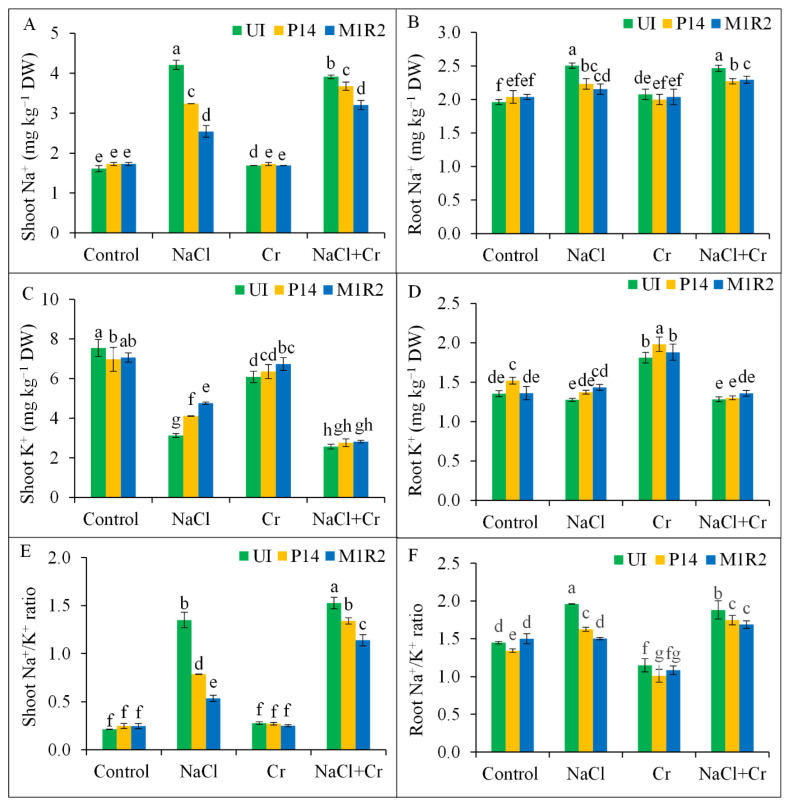
Effect of bacterial inoculation on ion homeostasis in rice plants (65 DAT) under NaCl, Cr, and NaCl + Cr stress conditions. Parameters measured include shoot Na^+^ (**A**), shoot K^+^ (**B**), root Na^+^ (**C**), root K^+^ (**D**), shoot Na^+^/K^+^ ratio (**E**), and root Na^+^/K^+^ ratio (**F**). Each column represents the mean ± standard deviation (SD) of three biological replicates. Error bars indicate the least significant difference (LSD) at a 5% level of significance. Different letters indicate statistically significant differences between treatments at *p* < 0.05, UI = Uninoculated.

Exposure to NaCl and combined NaCl + Cr stress disrupted ion homeostasis in rice plants by significantly increasing Na^+^ accumulation and decreasing K^+^ levels in both shoot and root tissues. This imbalance resulted in a sharp rise in the Na^+^/K^+^ ratio by 630 and 716% in shoots, and by 134 and 128% in roots, respectively, compared to the control (Figure 7A–F). Inoculation with PGPR strains P14 and M1R2 significantly reduced shoot Na^+^ content and enhanced shoot K^+^ content under both NaCl and NaCl + Cr stress conditions, leading to a partial restoration of the shoot Na^+^/K^+^ ratio (Figure 7A,C,E). Both strains also lowered root Na^+^ levels under NaCl and NaCl + Cr stress (Figure 7B). While a slight increase in root K^+^ content was observed under NaCl stress following inoculation, no significant effect was found under NaCl + Cr stress (Figure 7D,F). Consequently, both strains contributed to a reduction in the root Na^+^/K^+^ ratio under NaCl and NaCl + Cr stress compared to uninoculated controls. The most pronounced improvement in ion homeostasis, particularly the reduction of the shoot Na^+^/K^+^ ratio, was observed with strain M1R2 under both NaCl and NaCl + Cr stress. However, neither P14 nor M1R2 had any significant effect on Na^+^, K^+^, or Na^+^/K^+^ ratios under Cr stress alone (Figure 7A–F). Thus, HMT-PGPRs effectively retain ion homeostasis, which is essential for maintaining plant growth under both NaCl and NaCl + Cr stress conditions.

### 3.7. Evaluation of Inoculation of HMT-PGPR on Antioxidant Enzyme Activities

#### 3.7.1. Antioxidant Enzyme Activities

**Figure 8:**
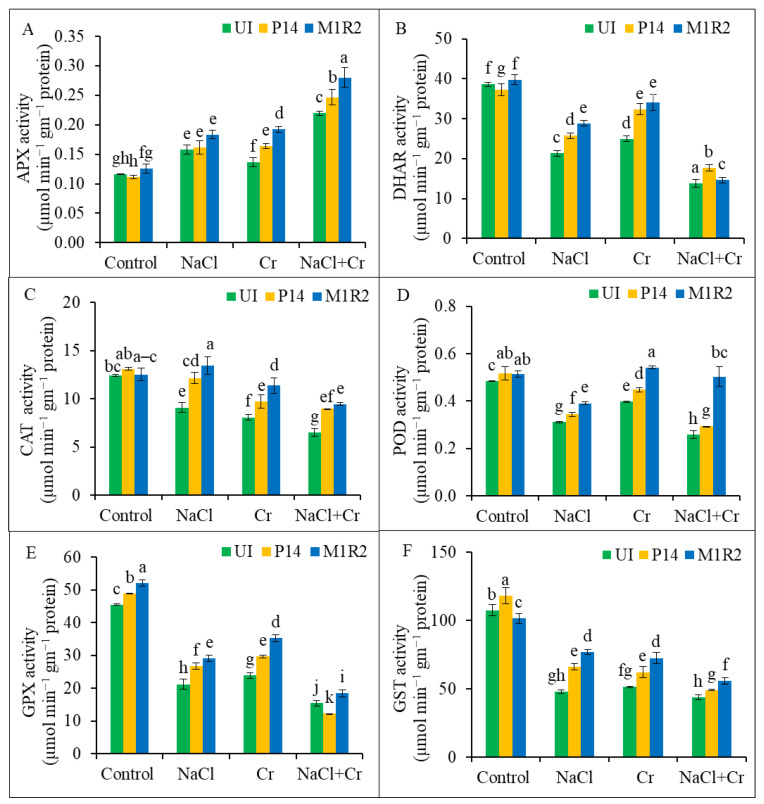
Effect of bacterial inoculation on the activities of antioxidant enzymes in rice plants (65 DAT) under NaCl, Cr, and NaCl + Cr stress conditions: (**A**) ascorbate peroxidase (APX), (**B**) dehydroascorbate reductase (DHAR), (**C**) catalase (CAT), (**D**) peroxidase (POD), (**E**) glutathione peroxidase (GPX), and (**F**) glutathione-*S*-transferase (GST). Each column represents the mean ± standard deviation (SD) of three biological replicates. Error bars indicate the least significant difference (LSD) at a 5% level of significance. Different letters indicate statistically significant differences between treatments at *p* < 0.05, UI = Uninoculated.

Rice plants exposed to NaCl, Cr, or combined NaCl + Cr stress showed a significant increase in APX activity, with the highest elevation (89%) observed under NaCl + Cr stress compared to control plants (Figure 8A). Inoculation with PGPR strains P14 and M1R2 further enhanced APX activity under all stress conditions, with M1R2 demonstrating superior performance compared to the respective uninoculated stressed plants. In contrast, DHAR activity declined significantly under NaCl, Cr, and NaCl + Cr stress by 45, 35, and 64%, respectively, relative to control plants (Figure 8B). However, inoculation with both PGPR strains markedly increased DHAR activity under all stress treatments, with M1R2 again showing the most pronounced enhancement. Similarly, CAT, POD, GPX, and GST activities were all reduced in uninoculated plants subjected to NaCl, Cr, or NaCl + Cr stress compared to unstressed controls (Figure 8C–F). Inoculation with either P14 or M1R2 significantly elevated the activities of these antioxidant enzymes under stress, with M1R2 consistently exhibiting greater improvement than P14. Therefore, PGPR-induced modulation of antioxidant capacity contributes to improved stress tolerance in plants.

#### 3.7.2. Glyoxalase System

**Figure 9:**
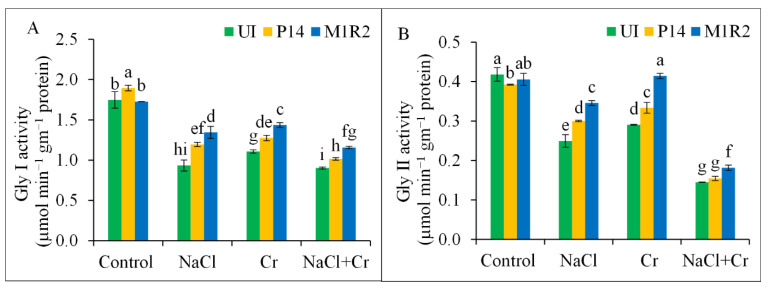
Effect of bacterial inoculation on Glyoxalase I (Gly I) activity (**A**) and Glyoxalase II (Gly II) activity (**B**) in rice plants (65 DAT) under NaCl, Cr, and combined NaCl + Cr stress conditions. Each column represents the mean ± standard deviation (SD) of three biological replicates. Error bars indicate the least significant difference (LSD) at a 5% level of significance. Different letters indicate statistically significant differences between treatments at *p* < 0.05, UI = Uninoculated.

The activities of glyoxalase enzymes Gly I and Gly II were significantly reduced in rice plants exposed to NaCl, Cr, or combined NaCl + Cr stress, with the most pronounced reductions observed under NaCl + Cr stress by 49 and 66%, respectively, compared to the control plants (Figure 9A,B). However, inoculation with PGPR strains P14 and M1R2 significantly restored Gly I and Gly II activities under all stress conditions. Notably, strain M1R2 outperformed P14 by enhancing Gly I activity by 44, 30, and 28%, and Gly II activity by 39, 43, and 26% under NaCl, Cr, and NaCl + Cr stress conditions, respectively, compared to the corresponding uninoculated stressed plants. Our results suggest that PGPR strains are capable of modulating the glyoxalase system in rice.

### 3.8. Effects of HMT-PGPR Inoculation on Cr Accumulation, Cr Translocation Factor, and Bioaccumulation Factor

**Figure 10:**
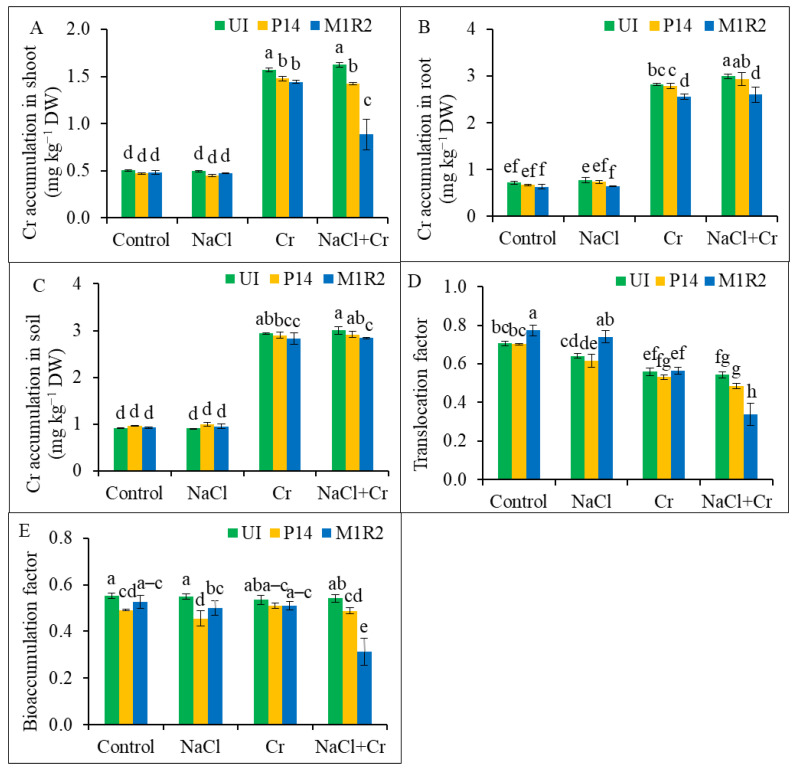
Effect of bacterial inoculation on chromium (Cr) accumulation in shoot (**A**), root (**B**), and soil (**C**); translocation factor (TF) (**D**) and bioaccumulation factor (BAF) (**E**) of rice plants (65 DAT) under NaCl, Cr, and combined NaCl + Cr stress conditions. Each column represents the mean ± standard deviation (SD) of three biological replicates. Error bars indicate the least significant difference (LSD) at a 5% level of significance. Different letters indicate statistically significant differences between treatments at *p* < 0.05, UI = Uninoculated.

In this study, PGPR inoculation did not exert a significant effect on shoot and root Cr concentrations under normal or salt stress conditions. Across plant organs, Cr accumulation was consistently higher in roots than in shoots. Specifically, shoots accumulated 1.57 mg kg^−1^ and 1.62 mg kg^−1^, while roots accumulated 2.81 mg kg^−1^ and 2.99 mg kg^−1^ under Cr and NaCl + Cr stress, respectively, indicating preferential Cr accumulation in roots (Figure 10A,B). However, PGPR strains P14 and M1R2 were able to reduce shoot Cr concentrations under Cr stress by 6 and 8%, respectively. Under combined NaCl + Cr stress, these reductions were more pronounced, including 13% with P14 and 46% with M1R2 inoculation (Figure 10A,B). A similar trend was observed in root Cr accumulation. M1R2 reduced root Cr content by 9% under Cr stress and 13% under NaCl + Cr stress. In contrast, P14 showed no significant effect on Cr accumulation in roots under either condition.

Additionally, M1R2 significantly decreased soil Cr concentrations by 4 and 5% under Cr and NaCl + Cr stress, respectively, compared to the respective controls (Figure 10C). This suggests that the endophytic strain M1R2 is more effective than the epiphytic P14 in reducing overall Cr accumulation in the rhizosphere–plant continuum, particularly under combined salt and HM stress. The translocation factor and BAF for Cr, indicating Cr movement from root to shoot and soil to root, respectively, are shown in Figure 10D,E. Under Cr stress, P14 inoculation slightly reduced both TF and BAF by 5%. M1R2 reduced BAF by 5% but had no significant effect on TF under Cr stress.

Under dual NaCl + Cr stress, both strains significantly reduced Cr translocation and accumulation: P14 reduced TF and BAF by 11 and 10%, respectively, while M1R2 inoculation achieved a substantial reduction 38% in TF and 42% in BAF. These results demonstrate that the endophytic M1R2 strain conferred the greatest reduction in Cr accumulation, translocation, and uptake efficiency under NaCl + Cr stress compared to the uninoculated stressed plants.

### 3.9. Principal Component Analysis

**Figure 11:**
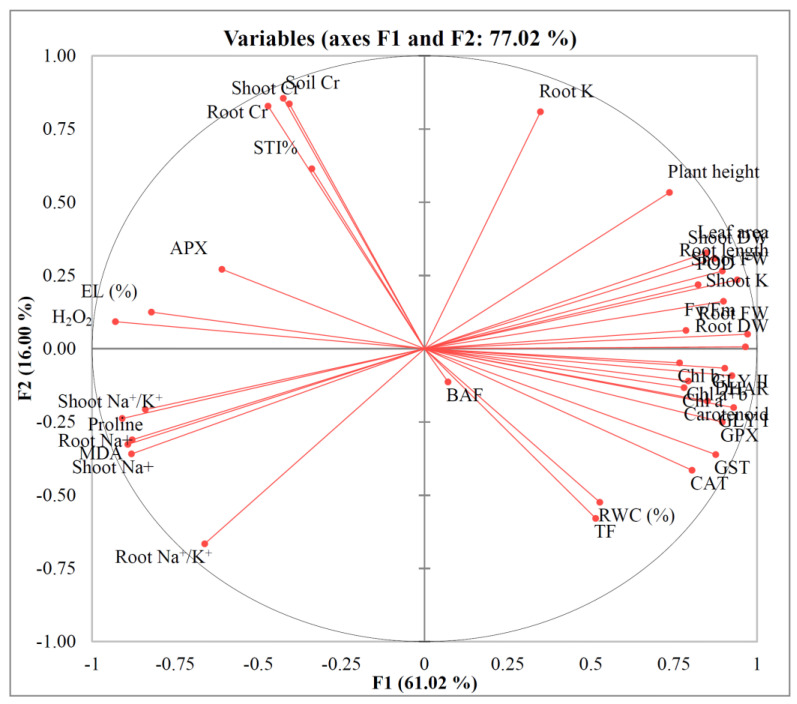
Principal component analysis (PCA) of different examined parameters. Here, H_2_O_2_: Hydrogen peroxide; MDA: Malondialdehyde; EL: Electrolyte leakage; Chl: Chlorophyll; RWC: Relative water content; APX: Ascorbate peroxidase; CAT: Catalase; DHAR: Dehydroascorbate reductase; GPX: Glutathione peroxidase; POD: Peroxidase; GST: Glutathione S-transferase; Gly I: Glyoxalase I; Gly II: Glyoxalase II; TF: Translocation factor; BAF: Bioaccumulation factor; STI%: Stress tolerance index.

The similarities among the various attributes examined in this study are illustrated in Figure 11. Most components associated with plant growth, photosynthetic pigments, antioxidant defense mechanisms, and the glyoxalase system were markedly influenced by the applied treatments. However, an exception was observed in the case of APX, which exhibited relatively stable activity and was not notably affected by the treatments.

## 4. Discussion

Plant growth-promoting rhizobacteria with salinity or HMT can enhance plant growth and development under individual or combined stress conditions by establishing mutualistic interactions with host plants [30,49]. Although the efficacy of *O. pseudogrignonense* and *A. woluwensis* in mitigating salinity or drought stress in various crop plants has been previously reported [28,29], the present study is the first to demonstrate the potential of these HMT-PGPR strains in alleviating NaCl, Cr, or combined NaCl + Cr stress in rice.

### 4.1. Inoculation of HMT-PGPR Improves Plant Growth and Biomass

In this study, NaCl, Cr, and combined NaCl + Cr stress conditions significantly reduced the biomass of rice plants, as evidenced by decreased FW and DW (Figure 2). Notably, inoculation with PGPR strains improved plant growth parameters under all stress conditions. These results suggest that PGPR strains can promote growth and enhance the stress tolerance of rice by alleviating oxidative stress induced by salinity and HMs (Figure 1 and Figure 2), aligning with the findings of Ji et al. [50] and Vishnupradeep et al. [49]. The PGPR stimulates plant growth through both direct mechanisms, such as the production of phytohormones [51], and indirect mechanisms like the mitigation of salt or metal stress [52]. Several studies have demonstrated that PGPR can produce siderophores, IAA, and ACC-deaminase under salt or Cr stress in plants, including rice, thereby improving growth and stress resilience [49,53,54,55,56]. Under stress conditions, PGPR-induced secretion of IAA also promotes root development, as reported by Yasmin et al. [57]. Furthermore, bacterial strains with ACC-deaminase activity lower stress-induced ethylene synthesis by converting ACC into ammonia and *α*-ketoglutarate, thereby supporting plant growth [58]. It may be mentioned that the stress resilience of the PGPR isolates could be attributed to the strong biofilm formation under salt stress by them; for example, *Stenotrophomonas* and *Bacillus* spp., underscoring their potential as plant growth-promoting agents for sustainable agriculture [59]. Therefore, the epiphytic strain P14 and the endophytic strain M1R2 appear to effectively enhance rice growth in salt-affected or Cr-contaminated soils.

### 4.2. Inoculation of HMT-PGPR Improves Photosynthetic Attributes

Salinity and Cr stress cause a significant decline in photosynthetic attributes (Figure 4A–D), primarily due to the inhibition of Chl biosynthesis enzymes, as reported by Karthik et al. [60]. Syed et al. [61] further demonstrated that ROS interference, modifications to thylakoid membrane components, and ultrastructural changes in DNA and proteins accelerate Chl degradation. However, the application of PGPRs under NaCl and Cr stress led to an increase in photosynthetic pigments (Figure 4), indicating that the photosynthetic efficiency of rice plants was improved. These findings align with the results of Ning et al. [56] and Alharby and Ali [62], where PGPR-treated rice plants exhibited higher Chl content under NaCl or Cr stress.

The PGPRs appear to promote Chl biosynthesis and pigment accumulation under stress conditions by enhancing water and nutrient uptake, elevating antioxidant compounds, and mitigating oxidative damage [63]. The PGPRs also facilitate the reduction of toxic Cr^6+^ to the less harmful Cr^3+^ via microbial metabolic processes, thereby enhancing Chl content in Cr-stressed plants [62]. According to Zafar-ul-Hye et al. [64], PGPR secretes phytohormones and growth regulators that further increase Chl levels. The observed increase in photosynthetic pigments corresponded well with physiological growth traits such as root length and biomass (Figure 1 and Figure 2).

The Chl fluorescence parameter F*_v_*/F*_m_* represents the maximum quantum efficiency of PSII. In this study, F*_v_*/F*_m_* values decreased under NaCl, Cr, and combined stress conditions (Figure 4E), indicating damage to the PS II system. However, PGPR-inoculated rice plants exhibited higher F*_v_*/F*_m_* values, suggesting that PGPRs help protect the photosynthetic machinery and improve Chl fluorescence, ultimately enhancing plant growth under stress, consistent with Siddika et al. [65].

### 4.3. Inoculation of HMT-PGPR Improves Relative Water Content and Osmotic Balance

NaCl, Cr, and combined NaCl + Cr stress significantly reduced leaf RWC in uninoculated rice plants, likely due to the loss of turgor pressure and impaired hydraulic conductance, thereby reducing growth [66]. However, PGPR inoculation increased RWC under stress (Figure 5A), suggesting improved stomatal regulation and root function. Similarly, Siddika et al. [65] found that the application of PGPR resulted in increased RWC under salt stress conditions. Plant growth-promoting rhizobacteria have been shown to enhance root growth and water uptake under salinity [67] and Cr stress [62]. Proline accumulation, commonly associated with osmotic adjustment, increased under NaCl, Cr, and NaCl + Cr stress (Figure 5B), as previously reported [64,67]. However, PGPR-inoculated plants exhibited reduced proline levels under stress, indicating improved osmotic regulation. Similar findings have demonstrated that inoculation with PGPR strains reduced the proline accumulation in plants under salt stress [65], Cr stress [68], and drought stress conditions [69]. This suggests that strains P14 and M1R2 may enhance stress tolerance by modulating the synthesis of compatible solutes, thereby maintaining osmotic balance [70].

During the entire vegetation period, plants experience multiple and continuous stresses. Proline accumulation is generally associated with osmotic stress, as it helps in osmoregulation by stabilizing proteins and cellular structures under stress conditions. However, in the case of Cr stress, particularly Cr(VI), the proline response does not directly correlate with osmotic adjustment. Instead, proline accumulation under Cr stress is more related to its role as a detoxifying agent, mitigating oxidative damage by reactive oxygen species (ROS). Therefore, while proline levels increase under Cr stress, this increase is primarily linked to oxidative stress and metabolic disruption, rather than a direct response to osmotic stress. As such, the combined effect of Cr and NaCl stress can indeed induce osmotic stress, as observed in this study.

### 4.4. Inoculation of HMT-PGPR Reduced Oxidative Damage

NaCl and Cr stress triggered elevated levels of oxidative stress markers—H_2_O_2_, MDA, and EL in rice plants. In contrast, PGPR inoculation reduced the accumulation of these markers (Figure 6A–C), consistent with previous studies [51,53,56,65,71]. The results suggest that PGPRs activate ROS-scavenging mechanisms, thereby mitigating oxidative damage under single and combined stress conditions (Figure 8 and Figure 9).

### 4.5. Inoculation of HMT-PGPR Improves Ion Homeostasis

Excessive Na^+^/K^+^ ratios disrupt cellular ion homeostasis, impair water uptake, and induce membrane damage [72]. In this study, PGPR-inoculated rice plants showed a reduced Na^+^/K^+^ ratio in both shoots and roots (Figure 7E,F), likely due to decreased Na^+^ uptake and enhanced K^+^ absorption (Figure 7A–D). This is consistent with previous findings [73,74].

The PGPRs may secrete EPS [58], which binds Na^+^ ions and reduces their bioavailability [75] while also mitigating HM toxicity via electrostatic interactions and redox mechanisms [76]. The PGPRs also produce siderophores and organic acids that chelate Cr, reducing its uptake and toxicity [54,77]. These processes help restore ion homeostasis and enhance tress tolerance.

### 4.6. Inoculation of HMT-PGPR Promotes the Modulation of Antioxidant Defense System

Stress-induced ROS accumulation and lipid peroxidation were alleviated by PGPR strains P14 and M1R2 (Figure 6A,B). The PGPR enhanced the activities of multiple antioxidant enzymes, including CAT, APX, POD, DHAR, GPX, and GST (Figure 8), which play vital roles in neutralizing ROS and maintaining redox homeostasis [55,78].

Although NaCl and Cr stress increased APX activity, they reduced CAT and POD activity, consistent with Ahmad et al. [79]. Plant growth-promoting rhizobacteria reversed these effects, enhancing antioxidant enzyme activities and supporting previous reports [80,81,82]. Elevated GST activity, essential for detoxifying ROS via GSH conjugation, was also observed [50,65]. Moreover, PGPRs activated glyoxalase enzymes (Gly I and Gly II), essential for detoxifying MG, a cytotoxic compound that accumulates under stress [42]. Stress reduced Gly I and Gly II activity (Figure 9), but PGPR inoculation restored their function, supporting findings from Alabdallah et al. [83] and Kapadia et al. [55].

### 4.7. Inoculation of HMT-PGPR Reduces Cr Concentrations and Its Bioaccumulation

Rice plants exposed to Cr and combined NaCl + Cr stress accumulated significantly more Cr in roots, shoots, and soil. However, treatment with the endophytic PGPR strain M1R2 markedly reduced Cr accumulation in these tissues (Figure 10A–C). This suggests that PGPR immobilized Cr in root tissues and restricted its upward translocation, possibly through mechanisms involving EPS, biofilms, siderophores, IAA, and protein secretion [84,85,86]. These compounds bind Cr ions, reduce bioavailability, and consequently decrease Cr bioaccumulation in plant tissues. In addition, Cr-tolerant strain “JS2” led to decreased Cr accumulation and significantly restricted Cr translocation to the above-ground parts of the plant [87]. Similarly, the lower translocation factor (TF) observed in plants inoculated with PGPR strain M1R2 indicates a reduced movement of Cr from roots to shoots. This limitation in Cr translocation may enhance the stress tolerance index (STI) (Figure 2F) by mitigating the toxic effects of both NaCl and Cr. These findings are consistent with those reported by Islam et al. [88]. In addition, endophytic bacteria M1R2 have to competitively colonize the plant interior, which is achieved using a battery of colonization traits, such as chemotaxis, flagella, lytic enzymes, and quorum sensing. Bacteria M1R2 respond to plant root exudates, followed by root surfaces and root interior colonization. Once inside, the competent endophytes can move to the aerial parts of the plants (stem and leaves).

### 4.8. Response to Epiphytic and Endophytic Strains

Epiphytic and endophytic strains are both plant-associated microbes; they colonize different niches and can have distinct or overlapping effects on plant health, growth, and stress tolerance. Epiphytes live on the surface of plant tissue (stems, roots), and endophytes reside in the internal tissues of plants without harming the host. They both help in plant growth promotion by producing phytohormones (e.g., auxins, gibberellins), solubilizing phosphates, or fixing atmospheric nitrogen. They also facilitate stress tolerance (e.g., drought, salinity) through the production of stress-response molecules like ACC deaminase and facilitate biocontrol activity by producing antimicrobial compounds. As both epiphytic and endophytic strains may possess overlapping functional genes involved in plant interaction and secondary metabolite production, especially when closely related genetically, their contribution to plants becomes similar. In other cases, effects diverge significantly, mainly due to colonization site and microenvironment. While endophytes operate within the plant, accessing internal cues and signals that usually span a long time, resulting in effective modulation of host immunity, the epiphytes face external environmental stresses and often have limited interaction with internal physiology. Hence, they produce localized responses or weak induced systemic resistance (ISR). The differential effects could be related to their genetic and functional response. Some strains, regardless of their niche, are naturally potent due to robust metabolite profiles or genetic versatility. The host (plant) genotype is another factor that could influence colonization efficiency and downstream physiological/biochemical responses.

## 5. Conclusions

This study demonstrated that NaCl, Cr, and combined NaCl + Cr stresses adversely impacted plant growth. However, inoculation with HMT-PGPR mitigated these negative effects by enhancing RWC, reducing levels of MDA, H_2_O_2_, proline, and EL, and modulating antioxidant enzyme activities. Consequently, PGPR inoculation improved rice performance under stress conditions, resulting in increased biomass and enhanced photosynthetic attributes. Notably, the endophytic PGPR strain M1R2 was particularly effective under combined NaCl + Cr stress, significantly reducing Na^+^ and Cr accumulation by lowering the Na^+^/K^+^ ratio and limiting Cr translocation from roots to shoots. These findings offer novel insights into plant–microbe interactions that enhance tolerance to salt and chromium stress and highlight the potential of HMT-PGPR for rehabilitating salt and Cr-contaminated soils. The PGPR demonstrates effective mitigation of salinity and chromium stress and could be employed for large-scale use in sustainable agriculture. Future research will evaluate the productivity of PGPR-inoculated rice under saline and chromium stress conditions to address global food security challenges.

## Data Availability

The original contributions presented in this study are included in the article. Further inquiries can be directed to the corresponding author.

## References

[1] Ullah H., Santiago-Arenas R., Ferdous Z., Attia A., Datta A. (2019). Improving water use efficiency, nitrogen use efficiency, and radiation use efficiency in field crops under drought stress: A review. Adv. Agron..

[2] Chung Y.S., Kim K.S., Hamayun M., Kim Y. (2019). Silicon confers soybean resistance to salinity stress through regulation of reactive oxygen and reactive nitrogen species. Front. Plant Sci..

[3] Phour M., Sindhu S.S., Parray J.A. (2023). Soil Salinity and Climate Change: Microbiome-Based Strategies for Mitigation of Salt Stress to Sustainable Agriculture BT. Climate Change and Microbiome Dynamics: Carbon Cycle Feedbacks.

[4] Jamil A., Riaz S., Ashraf M., Foolad M.R. (2011). Gene expression profiling of plants under salt stress. Crit. Rev. Plant Sci..

[5] Xiaoqin S., Dongli S., Yuanhang F., Hongde W., Lei G. (2021). Three-dimensional fractal characteristics of soil pore structure and their relationships with hydraulic parameters in biochar-amended saline soil. Soil Till. Res..

[6] Hashem A., Abd_Allah E.F., Alqarawi A.A., Al-Huqail A.A., Shah M.A. (2016). Induction of osmoregulation and modulation of salt stress in *Acacia gerrardii* Benth. by arbuscular mycorrhizal fungi and *Bacillus subtilis* (BERA 71). Biomed. Res. Int..

[7] Hasanuzzaman M., Nahar K., Alam M.M., Bhuyan M.B., Oku H., Fujita M. (2018). Exogenous nitric oxide pretreatment protects *Brassica napus* L. seedlings from paraquat toxicity through the modulation of antioxidant defense and glyoxalase systems. Plant Physiol. Biochem..

[8] Pan J., Peng F., Xue X., You Q., Zhang W., Wang T. (2019). The growth promotion of two salt-tolerant plant groups with PGPR inoculation: A meta-analysis. Sustainability.

[9] Krivokapic M. (2021). Study on the evaluation of (heavy) metals in water and sediment of skadar lake (Montenegro), with BCF assessment and translocation ability (TA) by *Trapa natans* and a review of SDGs. Water.

[10] Alam R., Rasheed R., Ashraf M.A., Hussain I., Ali S. (2023). Allantoin alleviates chromium phytotoxic effects on wheat by regulating osmolyte accumulation, secondary metabolism, ROS homeostasis and nutrient acquisition. J. Hazard. Mater..

[11] Suresh G., Ravichandran N., Ramesh B., Suresh A., Siva G.V. (2011). Isolation and characterization of chromium-tolerant bacteria from chromium containing waste water. Bioremed. Biodiver. Bioavail..

[12] Kaszycki P., Gabryś H., Appenroth K.-J., Jaglarz A., Sedziwy S., Walczak T., Kolochek H. (2005). Exogenously applied sulphate as a tool to investigate transport and reduction of chromate in the duckweed *Spirodela polyrhiza*. Plant Cell Environ..

[13] Su C.Q., Li L.Q., Yang Z.H., Chai L.Y., Liao Q., Shi Y., Li J.W. (2019). Cr (VI) reduction in chromium-contaminated soil by indigenous microorganisms under aerobic condition. Trans. Nonferrous Met. Soc. China.

[14] Smrithi A., Usha K. (2012). Isolation and characterization of chromium removing bacteria from tannery effluent disposal site. Intl. J. Adv. Biotec. Res..

[15] Singh H.P., Mahajan P., Kaur S., Batish D.R., Kohli R.K. (2013). Chromium toxicity and tolerance in plants. Environ. Chem. Lett..

[16] Mizan A., Mamun M.A.H., Islam M.S. (2023). Metal contamination in soil and vegetables around Savar tannery area, Dhaka, Bangladesh: A preliminary study for risk assessment. Heliyon.

[17] Vardharajula S., Zulfikar A.S., Grover M., Reddy G., Bandi V. (2011). Drought-tolerant plant growth promoting *Bacillus* spp., effect on growth, osmolytes, and antioxidant status of maize under drought stress. J. Plant Inter..

[18] Alqarawi A.A., Abd_Allah E.F., Hashem A. (2014). Alleviation of salt-induced adverse impact via mycorrhizal fungi in *Ephedra aphylla* Forssk. J. Plant Interact..

[19] Kumar V., Suryakant P., Kumar S., Kumar N. (2016). Effect of chromium toxicity on plants: A review. Agriways.

[20] Ma N.L., Lah W.A.C., Kadir N.A., Mustaqim M., Rahmat Z., Ahmad A., Lam S.D., Ismail M.R. (2018). Susceptibility and tolerance of rice crop to salt threat: Physiological and metabolic inspections. PLoS ONE.

[21] Basit F., Bhat J.A., Han J., Guan Y., Jan B.L., Shakoor A., Alansi S. (2022). Screening of rice cultivars for Cr-stress response by using the parameters of seed germination, morpho-physiological and antioxidant analysis. Saudi J. Biol. Sci..

[22] Paul D., Lade H. (2014). Plant-growth-promoting rhizobacteria to improve crop growth in saline soils: A review. Agron. Sustain. Dev..

[23] Agbodjato N.A., Babalola O.O. (2024). Promoting sustainable agriculture by exploiting plant growth-promoting rhizobacteria (PGPR) to improve maize and cowpea crops. PeerJ.

[24] Olanrewaju O.S., Glick B.R., Babalola O.O. (2017). Mechanisms of action of plant growth promoting bacteria. World J. Microbiol. Biotechnol..

[25] Dame Z.T., Rahman M., Islam T. (2021). Bacilli as sources of agrobiotechnology: Recent advances and future directions. Green Chem. Lett. Rev..

[26] Efe D. (2020). Potential plant growth-promoting bacteria with heavy metal resistance. Curr. Microbiol..

[27] Wang Y., Zhang G., Huang Y., Guo M., Song J., Zhang T., Long Y., Wang B., Liu H. (2022). A Potential biofertilizer—Siderophilic bacteria isolated from the rhizosphere of *Paris polyphylla* var. *yunnanensis*. Front. Microbiol..

[28] Saikia J., Sarma R.K., Dhandia R., Yadav A., Bharali R., Gupta V.K., Saikia R. (2018). Alleviation of drought stress in pulse crops with ACC deaminase producing rhizobacteria isolated from acidic soil of Northeast India. Sci. Rep..

[29] Khan M.A., Asaf S., Khan A.L., Adhikari A., Jan R., Ali S., Imran M., Kim K.-M., Lee I.-J. (2019). Halotolerant rhizobacterial strains mitigate the adverse effects of NaCl stress in soybean seedlings. BioMed Res. Int..

[30] Daraz U., Ahmad I., Li Q.S., Zhu B., Saeed M.F., Li Y., Ma J., Wang X.B. (2023). Plant growth promoting rhizobacteria induced metal and salt stress tolerance in *Brassica juncea* through ion homeostasis. Ecotoxicol. Environ. Saf..

[31] BRRI (Bangladesh Rice Research Institute) (2016). Adhunik Dhaner Chash.

[32] Shetty G., Hetrick D., Schwat P. (1995). Effects of mycorrhizal fertilizers amendments on zinc tolerance of plants. Environ. Pollut..

[33] Sairam R.K., Rao K.V., Srivastava G.C. (2002). Differential response of wheat genotypes to long term salinity stress relation to oxidative stress, antioxidant activity and osmolyte concentration. Plant Sci..

[34] Bates L.S., Waldren R.P., Teari D. (1973). Rapid determination of free proline for water stress studies. Plant Soil.

[35] Lichtenthaler H.K., Wellburn A.R. (1987). Chlorophylls and carotenoids: Pigments of photosynthetic biomembranes. Methods Enzymol..

[36] Yoshida S., Forno D.A., Cock J.H., Gomez K.A. (1976). Laboratory Manual for Physiological Studies of Rice.

[37] Campbell C.R., Plank C.O., Plank C.O. (1992). Sample preparation. Plant Analysis Reference Procedures for the Southern Region of the United States.

[38] Shahabivand S., Parvaneh A., Aliloo A.A. (2020). Different response of *Alyssum montanum* and *Helianthus annuus* to cadmium bioaccumulation mediated by the endophyte fungus *Serendipita indica*. Acta Ecol. Sin..

[39] Madhava Rao K.V., Sresty T.V.S. (2000). Antioxidative parameters in the seedlings of pigeon pea (*Cajanus cajan* L. Millapaugh) to Zn and Ni stresses. Plant Sci..

[40] Yu C.W., Murphy T.M., Lin C.H. (2003). Hydrogen peroxide induced chilling tolerance in mung beans mediated through ABA independent glutathione accumulation. Funct. Plant Biol..

[41] Dionisio-Sese M.L., Tobita S. (1998). Antioxidant responses of rice seedlings to salinity stress. Plant Sci..

[42] Hasanuzzaman M., Ahmed N., Saha T., Rahman M., Rahman K., Alam M.M., Rohman M.M., Nahar K. (2022). Exogenous salicylic acid and kinetin modulate reactive oxygen species metabolism and glyoxalase system to confer waterlogging stress tolerance in soybean (*Glycine max* L.). Plant Stress.

[43] Bradford M.M. (1976). A rapid and sensitive method for the quantitation of microgram quantities of protein utilizing the principle of protein-dye binding. Anal. Biochem..

[44] Hemeda H.M., Klein B.P. (1990). Effects of naturally occurring antioxidants on peroxidase activity of vegetable extracts. J. Food Sci..

[45] Nakano Y., Asada K. (1981). Hydrogen peroxide is scavenged by ascorbate specific peroxidase in spinach chloroplasts. Plant Cell Physiol..

[46] Elia A.C., Galarini R., Taticchi M.I., Dorr A.J.M., Mantilacci L. (2003). Antioxidant responses and bioaccumulation in *Ictalurus melas* under mercury exposure. Ecotoxicol. Environ. Saf..

[47] Yadav S.K., Singla-Pareek S.L., Ray M., Reddy M.K., Sopory S.K. (2005). Methylglyoxal levels in plants under salinity stress are dependent on glyoxalase I and glutathione. Biochem. Biophys. Res. Commun..

[48] Principato G.B., Rosi G., Talesa V., Giovanni E., Uotila L. (1987). Purification and characterization of two forms of glyoxalase II from the liver and brain of Wistar rats. Biochem. Biophys. Acta.

[49] Vishnupradeep R., Bruno L.B., Taj Z., Karthik C., Challabathula D., Tripti, Kumar A., Freitas H., Rajkumar M. (2022). Plant growth promoting bacteria improve growth and phytostabilization potential of *Zea mays* under chromium and drought stress by altering photosynthetic and antioxidant responses. Environ. Technol. Innov..

[50] Ji J., Zhang J., Wang X., Song W., Ma B., Wang R., Li T., Wang G., Guan C., Gao X. (2024). The alleviation of salt stress on rice through increasing photosynthetic capacity, maintaining redox homeostasis and regulating soil enzyme activities by *Enterobacter* sp. JIV1 assisted with putrescine. Microb. Res..

[51] Sultana S., Paul S.C., Parveen S., Alam S., Rahman N., Jannat B., Hoque S., Rahman M.T., Karim M.M. (2020). Isolation and identification of salt-tolerant plant growth-promoting rhizobacteria and its application for rice cultivation under salt stress. Can. J. Microbiol..

[52] Mallick I., Bhattacharyya C., Mukherji S., Dey D., Sarkar S.C., Mukhopadhyay U.K., Ghosh A. (2018). Effective rhizoinoculation and biofilm formation by arsenic immobilizing halophilic plant growth promoting bacteria (PGPB) isolated from mangrove rhizosphere: A step towards arsenic rhizoremediation. Sci. Total Environ..

[53] Sahoo R.K., Rani V., Tuteja N. (2021). *Azotobacter vinelandii* helps to combat chromium stress in rice by maintaining antioxidant machinery. 3 Biotech.

[54] Sultana S., Alam S., Karim M.M. (2021). Screening of siderophore-producing salt-tolerant rhizobacteria suitable for supporting plant growth in saline soils with iron limitation. J. Agric. Food. Res..

[55] Kapadia C., Patel N., Rana A., Vaidya H., Alfarraj S., Ansari M.J., Gafur A., Poczai P., Sayyed R.Z. (2022). Evaluation of plant growth-promoting and salinity ameliorating potential of halophilic bacteria isolated from saline soil. Front. Plant Sci..

[56] Ning Z., Lin K., Gao M., Han X., Guan Q., Ji X., Yu S., Lu L. (2024). Mitigation of salt stress in rice by the halotolerant plant growth-promoting bacterium *Enterobacter asburiae* D2. J. Xenobiot..

[57] Yasmin H., Nosheen A., Naz R., Bano A., Keyani R. (2017). *L*-tryptophan-assisted PGPR-mediated induction of drought tolerance in maize (*Zea mays* L.). J. Plant Interact..

[58] Jirakkakul J., Khoiri A.N., Duangfoo T., Dulsawat S., Sutheeworapong S., Petsong K., Wattanachaisaereekul S., Paenkaew P., Tachaleat A., Cheevadhanarak S. (2023). Insights into the genome of *Methylobacterium* sp. NMS14P, a novel bacterium for growth promotion of maize, chili, and sugarcane. PLoS ONE.

[59] Jhuma T.A., Rafeya J., Sultana S., Rahman M.T., Karim M.M. (2021). Isolation of endophytic salt-tolerant plant growth-promoting rhizobacteria from *Oryza sativa* and evaluation of their plant growth-promoting traits under salinity stress condition. Front. Sustain. Food Syst..

[60] Karthik C., Oves M., Thangabalu R., Sharma R., Santhosh S.B., Indra A.P. (2016). *Cellulosimicrobium funkei*-like enhances the growth of *Phaseolus vulgaris* by modulating oxidative damage under chromium (VI) toxicity. J. Adv. Res..

[61] Syed A., Elgorban A.M., Bahkali A.H., Eswaramoorthy R., Iqbal R.K., Danish S. (2023). Metal-tolerant and siderophore producing *Pseudomonas fluorescence* and *Trichoderma* spp. improved the growth, biochemical features and yield attributes of chickpea by lowering Cd uptake. Sci. Rep..

[62] Alharby H.F., Ali S. (2022). Combined role of Fe nanoparticles (Fe NPs) and *Staphylococcus aureus* L. in the alleviation of chromium stress in rice plants. Life.

[63] Cappellari L.D.R., Banchio E. (2020). Volatile organic compounds produced by *Bacillus amyloliquefaciens* GB03 ameliorate the effects of salt stress in *Mentha piperita* principally through acetoin emission. J. Plant Growth Regul..

[64] Zafar-ul-Hye M., Naeem M., Danish S., Khan M.J., Fahad S., Datta R., Brtnicky M., Kintl A., Hussain G.S., El-Esawi M.A. (2020). Effect of cadmium-tolerant rhizobacteria on growth attributes and chlorophyll contents of bitter gourd under cadmium toxicity. Plants.

[65] Siddika A., Rashid A.A., Khan S.N., Khatun A., Karim M.M., Prasad P.V.V., Hasanuzzaman M. (2024). Harnessing plant growth-promoting rhizobacteria, *Bacillus subtilis* and *B. aryabhattai* to combat salt stress in rice: A study on the regulation of antioxidant defense, ion homeostasis, and photosynthetic parameters. Front. Plant Sci..

[66] Tátrai Z.A., Sanoubar R., Pluhár Z., Mancarella S., Orsini F., Gianquinto G. (2016). Morphological and physiological plant responses to drought stress in *Thymus citriodorus*. Int. J. Agron..

[67] Wang G., Li B., Peng D., Zhao H., Lu M., Zhang L., Li J., Zhang S., Guan C., Ji J. (2022). Combined application of H_2_S and a plant growth promoting strain JIL321 regulates photosynthetic efficacy, soil enzyme activity and growth-promotion in rice under salt stress. Microbiol. Res..

[68] Tirry N., Kouchou A., El Omari B., Ferioun M., Ghachtouli E.N. (2021). Improved chromium tolerance of *Medicago sativa* by plant growth-promoting rhizobacteria (PGPR). J. Genet. Eng. Biotechnol..

[69] Nader A.A., Hauka F.I.A., Afify A.H., El-Sawah A.M. (2024). Drought-tolerant bacteria and arbuscular mycorrhizal fungi mitigate the detrimental effects of drought stress induced by withholding irrigation at critical growth stages of soybean (*Glycine max*, L.). Microorganisms.

[70] Ashraf M., Foolad M.R. (2007). Roles of glycine betaine and proline in improving plant abiotic stress resistance. Environ. Exp. Bot..

[71] Wang G., Zhang L., Zhang S., Li B., Li J., Wang X., Zhang J., Guan C., Ji J. (2023). The combined use of a plant growth promoting *Bacillus* sp. strain and GABA promotes the growth of rice under salt stress by regulating antioxidant enzyme system, enhancing photosynthesis and improving soil enzyme activities. Microbiol. Res..

[72] Zhao Z.J., Zhang H.L., Wang M.J., Zhang X.M., Li L.X. (2020). Salt stress-related regulation mechanism of intracellular pH and ion homeostasis in plants. Plant Physiol. J..

[73] Arora N.K., Fatima T., Mishra J., Mishra I., Verma S., Verma R., Verma M., Bhattacharya A., Verma P., Mishra P. (2020). Halo-tolerant plant growth promoting rhizobacteria for improving productivity and remediation of saline soils. J. Adv. Res..

[74] Asif S., Jan R., Kim N., Asaf S., Lubna, Khan M.A., Kim E.G., Jang Y.H., Bhatta D., Lee I.J. (2023). Halotolerant endophytic bacteria alleviate salinity stress in rice (*Oryza sativa* L.) by modulating ion content, endogenous hormones, the antioxidant system and gene expression. BMC Plant Biol..

[75] Akhtar S.S., Andersen M.N., Naveed M., Zahir Z.A., Liu F. (2015). Interactive effect of biochar and plant growth-promoting bacterial endophytes on ameliorating salinity stress in maize. Funct. Plant Biol..

[76] Dobrowolski R., Szcze A., Czemierska M., Jarosz-Wiko A.A. (2017). Studies of cadmium(II), lead(II), nickel(II), cobalt(II) and chromium(VI) sorption on extracellular polymeric substances produced by *Rhodococcus opacus* and *Rhodococcus rhodochrous*. Bioresour. Technol..

[77] Qin H., Wang Z., Sha W., Song S., Qin F., Zhang W. (2024). Role of plant-growth-promoting rhizobacteria in plant machinery for soil heavy metal detoxification. Microorganisms.

[78] Kanwal R., Maqsood M.F., Shahbaz M., Naz N., Zulfiqar U., Ali M.F., Jamil M., Khalid F., Ali Q., Sabir M.A. (2024). Exogenous ascorbic acid as a potent regulator of antioxidants, osmo-protectants, and lipid peroxidation in pea under salt stress. BMC Plant Biol..

[79] Ahmad S., Mfarrej M.F.B., El-Esawi M.A., Waseem M., Alatawi A., Nafees M., Saleem M.H., Rizwan M., Yasmeen T., Anayat A. (2024). Chromium-resistant *Staphylococcus aureus* alleviates chromium toxicity by developing synergistic relationships with zinc oxide nanoparticles in wheat. Ecotoxicol. Environ. Saf..

[80] Ali S., Waseem M., Hussain A., Rizwan M., Ahmad A., Khan N. (2021). Combined application of citric acid and Cr resistant microbes improved castor bean growth and photosynthesis while it alleviated Cr toxicity by reducing Cr^+6^ to Cr^3+^. Microorganisms.

[81] Nemat H., Shah A.A., Akram W., Ramzan M., Yasin N.A. (2020). Ameliorative effect of co-application of *Bradyrhizobium japonicum* EI09 and Se to mitigate chromium stress in *Capsicum annum* L.. Int. J. Phytoremediat..

[82] Sharma P., Chouhan R., Bakshi P., Gandhi S.G., Kaur R., Sharma A., Bhardwaj R. (2022). Amelioration of chromium-Induced oxidative stress by combined treatment of selected plant-growth-promoting rhizobacteria and earthworms via modulating the expression of genes related to reactive oxygen species metabolism in *Brassica juncea*. Front. Microbiol..

[83] Alabdallah N.M., Al-Shammari A.S., Saleem K., Alzahrani S.S., Raza A., Asghar M.A., Ullah A., Hussain M.I., Yong J.W.H. (2024). Unveiling the mechanisms of silicon-induced salinity stress tolerance in *Panicum turgidum*: Insights from antioxidant defense system and comprehensive metabolic and nutritional profiling. S. Afr. J. Bot..

[84] Rizvi A., Khan M.S. (2018). Heavy metal-induced oxidative damage and root morphology alterations of maize (*Zea mays* L.) plants and stress mitigation by metal tolerant nitrogen-fixing *Azotobacter chroococcum*. Ecotoxicol. Environ. Saf..

[85] Tirry N., Joutey N.T., Sayel H., Kouchou A., Bahafid W., Asri M., El Ghachtouli N. (2018). Screening of plant growth promoting traits in heavy metals resistant bacteria: Prospects in phytoremediation. J. Genet. Eng. Biotechnol..

[86] Mishra P., Mishra J., Arora N.K. (2021). Plant growth promoting bacteria for combating salinity stress in plants—Recent developments and prospects: A review. Microbiol. Res..

[87] Sarwar M.J., Shabaan M., Asghar H.N., Ayyub M., Ali Q., Zulfiqar U., Nazim M., Alarjani K.M., Elshikh M.S. (2023). Interaction of chromium (Cr) resistant plant growth promoting rhizobacteria with compost to phytostabilize Cr in spinach rhizosphere. Plant Stress.

[88] Islam M.K., Alam I., Khanam M.S., Lee S.Y., Waghmode T.R., Huh M.R. (2014). Accumulation and tolerance characteristics of chromium in nine jute varieties (“*Corchorus* spp.” and “*Hibiscus* spp.”). Plant Omics.

